# Clinical characteristics and clinical outcome of community clusters with SARS-CoV-2 infection

**DOI:** 10.3389/fpubh.2022.1010099

**Published:** 2023-01-09

**Authors:** Xueling Zhu, Wenrui Wu, Jianwen Ning, Tingting Dai, Daiqiong Fang, Jingjing Wu, Ding Shi

**Affiliations:** ^1^State Key Laboratory for Diagnosis and Treatment of Infectious Diseases, National Clinical Research Center for Infectious Diseases, Collaborative Innovation Center for Diagnosis and Treatment of Infectious Diseases, The First Affiliated Hospital, Zhejiang University School of Medicine, Hangzhou, China; ^2^Department of Emergency, The First Affiliated Hospital, College of Medicine, Zhejiang University, Hangzhou, China; ^3^Department of Endocrinology and Metabolism, The First Affiliated Hospital, Zhejiang University School of Medicine, Hangzhou, China

**Keywords:** COVID-19, SARS-CoV-2, clustering epidemic, clustered cases, sporadic cases

## Abstract

**Background:**

Community clustering is one of the main features of severe acute respiratory syndrome coronavirus 2 (SARS-CoV-2). However, few studies have been conducted on the clinical characteristics and clinical outcome of clustered cases and sporadic cases with COVID-19.

**Methods:**

We recruited 41 community clusters confirmed with SARS-CoV-2 infection compared with 49 sporadic cases in Zhejiang Province from 19 January 2020 to 9 June 2020. Clinical data were collected to evaluate the clinical outcome and characteristics of community clusters.

**Results:**

Compared to sporadic cases, clustered cases had significantly lower Acute Physiology and Chronic Health Evaluation II (APACHE II) score {5.0 [interquartile range (IQR), 2.0–7.5] vs. 7.0 [IQR, 4.0–12.5]; *P* = 0.005}, less members in intensive care unit (ICU) (6 [14.6%] vs. 18 [36.7%]; *P* = 0.018), and shorter time of viral shedding in fecal samples (18.5 [IQR, 17.0–28.3] vs. 32.0 [IQR, 24.3–35.5]; *P* = 0.002). Univariable logistic regression revealed that older age (odds ratios 1.078, 95% confidence intervals 1.007–1.154, per year increase; *p* = 0.032), high APACHE II score (3.171, 1.147–8.76; *P* = 0.026), elevated interleukin-2 levels (3.078, 1.145–8.279; *P* = 0.026) were associated with ICU admission of clustered cases.

**Conclusions:**

Compared to sporadic cases, clustered cases exhibited milder disease severity and a better clinical outcome, which may be closely related to the management of early detection, early diagnosis, early treatment and early isolation of COVID-19.

## Introduction

There was an outbreak of an unexplained pneumonia in Wuhan, Hubei Province, China in December 2019 (1, 2). The virus has been isolated from infected patients and was named severe acute respiratory syndrome coronavirus 2 (SARS-CoV-2; previously known as 2019-nCoV) by 7 January 2020 (3). The disease was officially named coronavirus disease 2019 (COVID-19) by the WHO in February 2020 (3). Numerous clinical studies on COVID-19 have been published, including epidemiology, clinical features, laboratory findings, imaging features, risk factors, and clinical outcome (3–6). Chan et al. were the first to publish a study of a family cluster, noting the existence of person-to-person transmission of SARS-CoV-2 and that family clustering was the main characteristic of COVID-19 (7). A retrospective study of clustered cases and sporadic cases in Wuhan showed that the incidence of COVID-19 was significantly increased in cluster-onset families compared with solitary-onset families (8). In addition, older age and an elevated neutrophil/lymphocyte ratio are the main risk factors for death in infected patients in cluster-onset families (8). However, there are still few comparative studies assessing clustered cases and sporadic cases.

Hence, we conducted a study on 90 hospitalized patients with confirmed SARS-CoV-2 infection in Zhejiang Province. We compared the clinical characteristics between clustered cases and sporadic cases during hospitalization to evaluate the differences in the impacts of different clinical characteristics between these two case types.

## Methods

### Data sources

We conducted a retrospective study to investigate 90 patients confirmed with COVID-19 infection admitted to the First Affiliated Hospital, Zhejiang University School of Medicine, Zhejiang Province, China.

The entry criteria included the following: (1) patients with COVID-19 infection were confirmed once admitted, based on real-time reverse transcription-polymerase chain reaction (RT-PCR) assay of nasopharyngeal swab specimens by the laboratory of the First Affiliated Hospital, Zhejiang University School of Medicine, Zhejiang Province, China; (2) patients were discharged by June 30, 2020.

The exclusion criteria included: (1) missing case information; (2) missing laboratory test results.

Information on all patients was collected, including demographic data, body mass index, comorbidities, clinical symptoms, exposure history, laboratory findings, treatment, and clinical outcomes. Comorbidities included hypertension, diabetes mellitus, and fatty liver disease. Laboratory findings included complete blood count, coagulation profile, liver and renal function, creatine kinase, lactate dehydrogenase, electrolytes, myocardial enzymes, C-reactive protein (CRP), inflammatory factors, complements, and immunoglobulins. Chest computed tomography (CT) scans were performed. Treatment included antiviral therapy, hormonal therapy, antibiotics, mechanical ventilation, extracorporeal membrane oxygenation (ECMO), artificial liver support system (ALSS), and continuous renal replacement therapy (CRRT).

### Definition

Patients were divided into moderate, severe, or critical types according to the *Diagnosis and Treatment Protocol for Novel Coronavirus Pneumonia (Trial Version 9)* (9). Moderate cases were defined as having fever and respiratory symptoms and radiological findings of pneumonia. Severe adult cases were defined as having dyspnea (respiratory rate ≥ 30 breaths/min), resting oxygen saturation ≤ 93%, arterial oxygen partial pressure/fraction of inspired oxygen ≤ 300 mmHg (1 mmHg = 0.133 kPa), or cases with chest imaging showing obvious lesion progression within 24–48 h >50%. Critical cases were defined based on respiratory failure and the requirement of mechanical ventilation, shock, or other organ failure and the requirement for intensive care unit (ICU) care.

According to the *Protocol on Prevention and Control of Novel Coronavirus Pneumonia (Edition 9)*, exposure history referred to close contact with a COVID-19-infected individual (with positive results based on the nucleic acid test) within 2 weeks prior to the onset of disease. Clustered cases were defined as two or more cases with fever and/or respiratory symptoms in a small area, such as families, offices, and schools within 2 weeks, including index cases and secondary cases. Sporadic cases were defined as only one case with fever and/or symptoms in the small area.

Fever was defined as an axillary temperature of 37.4°C or greater. Fever duration was defined as the time from the onset of fever to the return of normal temperature. Time of viral shedding was the duration of positive RT-PCR result of specimens. The Acute Physiology and Chronic Health Evaluation (APACHE) II scores were calculated as described in the published literatures (10). Clinical outcomes were defined as the comprehensive assessment of clinical features, including clinical grades, laboratory findings, ICU care and so on.

### Statistical analysis

Data were statistically analyzed and plotted using Excel (Microsoft Corporation, Redmond, Washington, USA), SPSS 26.0 software (IBM, Armonk, New York, USA) and GraphPad Prism 8.0 software (GraphPad Software, San Diego, California, USA). Data were analyzed for normal distribution and homogeneity of variance before statistical analysis. Normally distributed continuous variables were expressed as the mean with standard deviation (mean ± sd). Non-normally distributed continuous variables were expressed as median with interquartile range (IQR). Categorical variables were expressed as numbers and percentages. Comparisons between groups of normally distributed continuous variables used parametric *t*-tests. Comparisons between groups of non-normally distributed measures used non-parametric Mann-Whitney *U*-tests. Comparisons between groups of categorical variables used χ^2^ test. Univariable logistic regression analysis was used to investigate the risk factors for ICU admission in clustered cases, and odds ratios with 95% confidence intervals were calculated. *P*-value < 0.05 (two-tailed) suggested that the difference was statistically significant.

### Ethical approval

This study was approved by the Ethics Committee of the First Affiliated Hospital, Zhejiang University School of Medicine.

## Results

### Patients' characteristics

From 19 January 2020 to 9 June 2020, 90 admitted hospital patients were confirmed with SARS-CoV-2 infection in The First Affiliated Hospital, Zhejiang University School of Medicine. In total, 41 were clustered cases, and 49 were sporadic cases. The average age of all patients was 52.6 ± 15.9 years. In total, 55 (61.1%) of the infected patients were male.

In total, 66 (73.3%) patients had exposure history. The proportion of definite exposure history was increased in clustered cases compared with sporadic cases (38 [92.7%] vs. 28 [57.1%]; *P* < 0.001).

In total, 53 (58.9%) of the infected patients had underlying diseases. Compared with sporadic cases, clustered cases had less percentage of comorbidities (19 [46.3%] vs. 34 [69.4%]; *P* = 0.027), such as hypertension (8 [19.5%] vs. 22 [44.9%]; *P* = 0.011).

The most common symptoms at onset of illness were fever (75 [83.4%]), cough (74 [82.2%]), and expectoration (43 [47.8%]). In clustered cases, patients were less likely to have headache (1 [2.4%] vs. 7 [14.3%]; *P* = 0.049) and myalgia (7 [17.1%] vs. 13 [26.5%]; *P* = 0.048). However, no difference in symptoms were noted between two groups of patients with the exception of headache and myalgia (see in Table 1; Supplementary Table 1).

**Table 1:** Clinical characteristics and laboratory findings of 90 patients with laboratory confirmed coronavirus disease 2019 infection.

	**All patients (*n* = 90)**	**Clustered cases (*n* = 41)**	**Sporadic cases (*n* = 49)**	***P*-value**
**Clinical characteristics**				
Age, years	52.6 ± 15.9	50.0 ± 17.7	54.8 ± 14.1	0.153
Gender				0.372
Male	55, 61.1%	23, 56.1%	32, 65.3%	
Female	35, 38.9%	18, 43.9%	17, 34.7%	
Exposure history	66, 73.3%	38, 92.7%	28, 57.1%	< 0.001^***^
Any coexisting disorder	53, 58.9%	19, 46.3%	34, 69.4%	0.027^*^
Hypertension	30, 33.3%	8, 19.5%	22, 44.9%	0.011^*^
**Signs and symptoms**				
Headache	8, 8.9%	1, 2.4%	7, 14.3%	0.049^*^
Myalgia	20, 22.2%	7, 17.1%	13, 26.5%	0.048^*^
**Laboratory findings**				
Prothrombin time (s)	11.8 (11.3–12.3)	11.6 (11.3–12.1)	11.9 (11.4–12.6)	0.045^*^
Glomerular filtration rate (mL/min)	94.0 (72.6–104.9)	96.4 (77.2–110.7)	89.1 (66.1–100.7)	0.028^*^
Lactate dehydrogenase (U/L)	245.5 (196.0–336.3)	223.0 (194.0–306.5)	274.0 (219.0–356.0)	0.037^*^
Interleukin-4 (pg/mL)	1.8 (1.4–1.8)	1.4 (1.0–1.8)	1.8 (1.4–1.8)	0.011^*^
Tumor necrosis factor-α (pg/mL)	16.5 (6.7–50.4)	11.2 (6.5–44.6)	22.1 (12.2–65.0)	0.023^*^

^*^0.01 < *P* < 0.05; ^***^P ≤ 0.001.

### Laboratory findings

The trend in laboratory findings at admission was approximately the same in sporadic and clustered cases. Only some differences in laboratory findings were noted between clustered cases and sporadic cases. The mean glomerular filtration rate (GFR) was significantly increased in clustered cases than sporadic cases (96.4 [IQR 77.2–110.7] vs. 89.1 [IQR 66.1–100.7]; *P* = 0.028). Lactate dehydrogenase levels were significantly reduced in clustered cases compared with sporadic cases (223.0 [IQR, 194.0–306.5] vs. 274.0 [IQR, 219.0–356.0]; *P* = 0.037).

In terms of inflammatory cytokines, the levels of interleukin-4 (IL-4) (1.4 [IQR 1.0–1.8] vs. 1.8 [IQR 1.4–1.8]; *P* = 0.011) and Tumor Necrosis Factor-α (11.2 [IQR 6.5–44.6] vs. 22.1 [IQR 12.2–65.0]; *P* = 0.023) were significantly reduced in clustered cases compared with sporadic cases (see Table 1; Supplementary Table 2).

### Clinical treatments

Eighty-nine (98.9%) patients received antiviral therapy, and 70 (77.8%) patients received corticosteroids. Time from illness onset to the initiation of antiviral therapy was shorter in clustered cases compared with sporadic cases (5.0 [IQR, 2.0–7.0] vs. 7.0 [IQR, 4.3–10.0]; *P* = 0.002). Antiviral therapy included single or combined use of abidol, lopinavir/ritonavir, and alpha-interferon. In addition, time from illness onset to the use of corticosteroids and to the end of intravenous corticosteroids use in clustered cases were both shorter compared with sporadic cases (7.0 [IQR, 4.5–8.0] vs. 8.0 [IQR, 7.0–11.0], *P* = 0.003; 17.5 [IQR, 13.8–21.3] vs. 21.0 [IQR, 18.0–24.0], *P* = 0.010, respectively).

A lower number of patients in clustered cases received treatments, such as antibiotics, mechanical ventilation, and ALSS, compared with sporadic cases (14 [34.1%] vs. 29 [59.2%], *P* = 0.018; 0 [0.0%] vs. 8 [16.3%], *P* = 0.019; 0 [0.0%] vs. 10 [20.4%], *P* = 0.006, respectively) (see Table 2).

**Table 2:** Clinical treatments of 90 patients with laboratory confirmed coronavirus disease 2019 infection.

**Treatments**	**All patients (*n* = 90)**	**Clustered cases (*n* = 41)**	**Sporadic cases (*n* = 49)**	***P*-value**
Antiviral therapy	89, 98.9%	41, 100.0%	48, 98.0%	0.358
Time from illness onset to the initiation of antiviral therapy, days	5.0 (4.0–8.0)	5.0 (2.0–7.0)	7.0 (4.3–10.0)	0.002^**^
Time from illness onset to the end of antiviral therapy, days	21.0 (117.5–27.5)	20.0 (17.0–25.0)	23.0 (18.0–30.8)	0.111
Use of corticosteroids	70, 77.8%	31, 75.6%	39, 79.6%	0.651
Time from illness onset to the use of corticosteroids, days	8.0 (6.0–9.0)	7.0 (4.5–8.0)	8.0 (7.0–11.0)	0.003^**^
Time from illness onset to the end of intravenous corticosteroids use, days	19.5 (16.8–24.0)	17.5 (13.8–21.3)	21.0 (18.0–24.0)	0.010^**^
Ambroxol	56, 62.2%	21, 51.2%	35, 71.4%	0.049^*^
Antibiotic therapy	43, 47.8%	14, 34.1%	29, 59.2%	0.018^*^
Use of gamma globulin	36, 40.0%	14, 34.1%	29, 59.2%	0.863
Use of probiotics	9, 10.0%	5, 12.2%	4, 8.2%	0.778
Use of vasopressors	2, 2.2%	0, 0.0%	2, 4.1%	0.555
Mechanical ventilation	8, 8.9%	0, 0.0%	8, 16.3%	0.019^*^
ECMO	6, 6.7%	0, 0.0%	6, 12.2%	0.058
ALSS	10, 11.1%	0, 0.0%	10, 20.4%	0.006^**^
CRRT	5, 5.6%	0, 0.0%	5, 10.2%	0.100

ECMO, extracorporeal membrane oxygenation; ALSS, artificial liver support system; CRRT, continuous renal replacement therapy. ^*^0.01 < *P* < 0.05; ^**^0.001 < *P* ≤ 0.01.

### Clinical outcomes

The median time from illness onset to confirmed diagnosis was shorter in clustered cases than sporadic cases (4.0 [IQR, 1.0–6.0] vs. 6.0 [IQR, 4.0–7.0]; *P* = 0.032). The length of stay of all patients was 16.5 (IQR, 13.0–24.0) days. Compared to sporadic cases, clustered cases had significantly lower APACHE II score (5.0 [IQR, 2.0–7.5] vs. 7.0 [IQR, 4.0–12.5]; *P* = 0.005) on the day of hospital admission. The proportion of critical illness (7 [17.1%] vs. 18 [36.7%]; *P* = 0.038) and ICU care (6 [14.6%] vs. 18 [36.7%]; *P* = 0.018) was reduced in clustered cases compared with sporadic cases.

**Figure 1:**
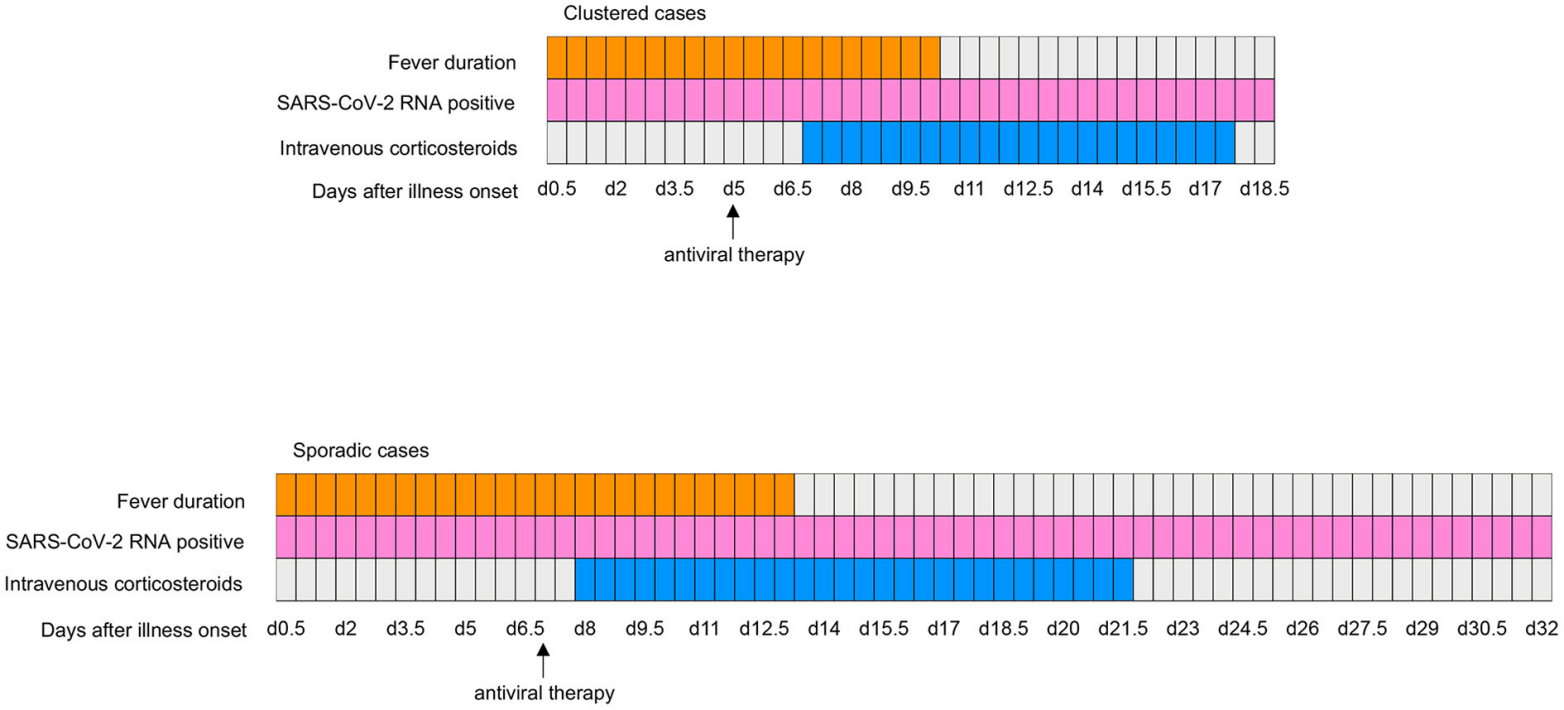
Clinical courses of major symptom and treatments and duration of viral shedding from illness onset in patients hospitalized with COVID-19. SARS-CoV-2, severe acute respiratory syndrome coronavirus 2; COVID-19, coronavirus disease 2019.

We detected acute respiratory distress syndrome (ARDS), bacterial infection, intestinal flora disorders and fecal RNA positivity in 10 (11.1%), 16 (17.8%), 9 (10.0%), and 30 (35.5%) patients, respectively. Compared with sporadic cases, clustered cases were less likely to develop ARDS (0 [0%] vs. 10 [20.4%]; *P* = 0.006) and bacterial infection (3 [7.3%] vs. 13 [26.5%]; *P* = 0.036). Fever persisted for a shorter time in clustered cases compared with sporadic cases (10.0 [IQR, 6.0–12.8] vs. 13.0 [IQR, 9.0–27.0]; *P* = 0.020). Shortened viral shedding in fecal samples was observed in clustered cases compared with sporadic cases (18.5 [IQR, 17.0–28.3] vs. 32.0 [IQR, 24.3–35.5]; *P* = 0.002) (see Table 3; Figure 1).

**Table 3:** Clinical outcomes of 90 patients with laboratory confirmed coronavirus disease 2019 infection.

**Outcomes**	**All patients (*n* = 90)**	**Clustered cases (*n* = 41)**	**Sporadic cases (*n* = 49)**	***P*-value**
LOS	16.5 (13.0–24.0)	16.0 (12.5–20.0)	19.0 (13.5–27.5)	0.087
APACHE II	6.0 (3.0–11.0)	5.0 (2.0–7.5)	7.0 (4.0–12.5)	0.005^**^
Time from illness onset to disease diagnosis, days	5.0 (2.0–7.0)	4.0 (1.0–6.0)	6.0 (4.0–7.0)	0.032^*^
Fever duration, days	11.0 (8.0–18.0)	10.0 (6.0–12.8)	13.0 (9.0–27.0)	0.020^*^
Duration of viral shedding in fecal samples, days	23.5 (18.0–32.3)	18.5 (17.0–28.3)	32.0 (24.3–35.5)	0.002^**^
Duration of viral shedding in the sputum sample, days	17.0 (13.0–24.3)	17.0 (13.0–21.5)	17.0 (13.0–28.5)	0.336
**Complications**				
ARDS	10, 11.1%	0, 0%	10, 20.4%	0.006^**^
Bacterial infection	16, 17.8%	3, 7.3%	13, 26.5%	0.036^*^
Intestinal flora disorders	9, 10.0%	5, 12.2%	4, 8.2%	0.778
Fecal RNA positivity	30, 35.3%	18, 47.4%	12, 25.5%	0.054
**Clinical grade**				0.038^*^
Moderate and severe	65, 72.2%	34, 82.9%	31, 63.3%	
Critical	25, 27.8%	7, 17.1%	18, 36.7%	
ICU care	24, 26.7%	6, 14.6%	18, 36.7%	0.018^*^
Time from illness onset to the improvement in chest CT scans manifestations, days	14.0 (12.0–19.0)	14.0 (11.8–18.0)	14.5 (12.0–20.0)	0.434

LOS, length of stay; APACHE II, acute physiology and chronic health evaluation II; ARDS, acute respiratory distress syndrome; ICU, intensive care unit; CT, computed tomography. ^*^0.01 < *P* < 0.05; ^**^0.001 < *P* ≤ 0.01.

### Difference in clustered cases admitted or not admitted to ICU

Of the clustered cases, 6 (14.6%) were admitted to the ICU. Patients with ICU care were older than those with non-ICU care (66.5 ± 18.2 vs. 47.2 ± 16.2; *P* = 0.012). Compared with non-ICU patients, ICU patients were more likely to have comorbidities (5 [83.3%] vs. 14 [40.0%]; *P* = 0.049), to exhibit an increased APACHE II score (12.5 [IQR, 9.8–14.3] vs. 3.0 [IQR, 2.0–6.0]; *P* < 0.001) at admission, to develop bacterial infection (2 [33.3%] vs. 1 [2.9%]; *P* = 0.008), and to be treated with antibiotics (6 [100.0%] vs. 8 [22.9%]; *P* < 0.001).

White blood cell (WBCs) count, and neutrophil count at admission were increased, while GFR at admission were reduced in ICU cases (10.1 [IQR, 8.4–18.5] vs. 5.2 [IQR, 3.9–7.8], *P* = 0.007; 9.1 [IQR, 7.6–16.6] vs. 3.7 [IQR, 2.3–6.7], *P* = 0.005; 80.5 [IQR, 60.8–94.3] vs. 99.7 [IQR, 83.2–112.5], *P* = 0.015, respectively). Blood glucose, troponin I (TNI), IL-2, and immunoglobulin G (IgG) levels were increased substantially in ICU patients compared with non-ICU patients (8.1 [IQR, 7.5–12.9] vs. 6.3 [IQR, 4.7–7.9], *P* = 0.022; 0.016 [IQR, 0.007–0.020] vs. 0.003 [IQR, 0.002–0.006], *P* = 0.012; 2.0 [IQR, 1.0–3.2] vs. 1.0 [IQR, 0.5–1.2], *P* = 0.029; 2,192.5 [IQR, 1,645.3–2,897.3] vs. 1,233.0 [IQR, 1,015.0–1,805.5], *P* = 0.004, respectively).

The improvement in chest CT scans occurred much later in ICU patients compared with non-ICU patients (23.0 [IQR, 20.0–28.5] vs. 13.0 [IQR, 11.0–17.0]; *P* = 0.001) (see Table 4).

**Table 4:** Difference in clustered cases admitted or not admitted to ICU.

	**Admitted to ICU**	**Not admitted to ICU**	***P*-value**
Age	66.5 ± 18.2	47.2 ± 16.2	0.012^*^
Any coexisting disorder	5, 83.3%	14, 40.0%	0.049^*^
Disease severity status			< 0.001^***^
Moderate and severe	0, 0.0%	34, 97.1%	
Critical	6, 100.0%	1, 2.9%	
APACHE II	12.5 (9.8–14.3)	3.0 (2.0–6.0)	< 0.001^***^
Bacterial infection	2, 33.3%	1, 2.9%	0.008^**^
Antibiotics	6, 100.0%	8, 22.9%	< 0.001^***^
Time from illness onset to the improvement in chest CT scans manifestations, days	23.0 (20.0–28.5)	13.0 (11.0–17.0)	0.001^***^
White blood cell count (× 10^9^/L)	10.1 (8.4–18.5)	5.2 (3.9–7.8)	0.007^**^
Neutrophil count (× 10^9^/L)	9.1 (7.6–16.6)	3.7 (2.3–6.7)	0.005^**^
Glomerular filtration rate (mL/min)	80.5 (60.8–94.3)	99.7 (83.2–112.5)	0.015^*^
Blood glucose (mmol/L)	8.1 (7.5–12.9)	6.3 (4.7–7.9)	0.022^*^
Troponin I (ng/mL)	0.016 (0.007–0.020)	0.003 (0.002–0.006)	0.012^*^
Interleukin-2 (pg/mL)	2.0 (1.0–3.2)	1.0 (0.5–1.2)	0.029^*^
Immunoglobulin G (mg/dL)	2,192.5 (16,45.3–2,897.3)	1,233.0 (1,015.0–1,805.5)	0.004^**^

ICU, intensive care unit; APACHE II, acute physiology and chronic health evaluation II; CT, computed tomography. ^*^0.01 < *P* < 0.05; ^**^0.001 < *P* ≤ 0.01; ^***^P ≤ 0.001.

### Difference in sporadic cases admitted or not admitted to ICU

We compared the differences in sporadic cases admitted or not admitted to ICU (see Table 5) to figure out the risk factors of ICU admission between the two groups. Many indicators were significantly different between patients admitted or not admitted to ICU in both sporadic cases and clustered cases, such as age, APACHE II score, WBCs count, neutrophil count, GFR, TNI, and IgG. There were some indicators that differed significantly only in sporadic cases, such as Albumin, Hydroxybutyrate dehydrogenase, CRP, Procalcitonin, IL-6, and IL-10.

**Table 5:** Difference in sporadic cases admitted or not admitted to ICU.

	**Admitted to ICU**	**Not admitted to ICU**	***P*-value**
Age	63.2 ± 14.8	50.0 ± 11.3	0.003^**^
Disease severity status			< 0.001^***^
Moderate and severe	0, 0.0%	31, 100.0%	
Critical	18, 100.0%	0, 0.0%	
LOS	27.5 (15.0–42.3)	15.0 (11.0–23.0)	0.003^**^
APACHE II	13.0 (12.0–16.5)	5.0 (2.0–7.0)	< 0.001^***^
Antibiotics	15, 83.3%	14, 45.2%	0.009^**^
Use of gamma globulin	13, 72.2%	7, 22.6%	0.001^***^
Fever duration, days	32.5 (13.0–46.0)	10.0 (7.0–13.0)	< 0.001^***^
Time from illness onset to the improvement in chest CT scans manifestations, days	19.0 (15.0–57.0)	13.0 (11.5–16.5)	0.001^***^
Time from illness onset to the end of antiviral therapy, days	28.0 (23.5–34.0)	19.0 (16.0–29.0)	0.004^**^
Time from illness onset to the end of intravenous corticosteroids use, days	23.0 (20.5–38.0)	18.0 (17.0–22.0)	0.002^**^
PO_2_/FIO_2_	167.4 (91.8–276.3)	321.9 (250.5–403.5)	0.011^*^
White blood cell count (× 10^9^/L)	7.9 (4.5–13.2)	5.1 (3.0–8.0)	0.032^*^
Neutrophil count (× 10^9^/L)	7.0 (3.5–12.0)	3.5 (1.7–6.6)	0.003^**^
Albumin (g/L)	33.6 (31.0–36.9)	41.5 (35.6–44.2)	< 0.001^***^
Glomerular filtration rate (mL/min)	66.1 (53.2–100.4)	91.4 (79.9–102.4)	0.031^*^
Hydroxybutyrate dehydrogenase (U/L)	335.5 (296.8–420.0)	228.0 (187.0–283.0)	0.001^***^
Troponin I (ng/mL)	0.006 (0.002–0.028)	0.003 (0.001–0.006)	0.036^*^
C-reactive protein (mg/L)	50.6 (26.2–96.1)	16.2 (4.6–35.3)	< 0.001^***^
Procalcitonin (ng/mL)	0.11 (0.07–0.32)	0.05 (0.03–0.07)	< 0.001^***^
Interleukin-6 (pg/mL)	57.1 (24.1–148.6)	13.6 (7.4–51.2)	0.005^**^
Interleukin-10 (pg/mL)	6.7 (5.4–9.0)	3.5 (2.9–6.4)	0.009^**^
Immunoglobulin G (mg/dL)	1,488.0 (1,138.5–2,189.0)	1,231.0 (983.8–1,401.8)	0.031^*^

ICU, intensive care unit; LOS, length of stay; APACHE II, acute physiology and chronic health evaluation II; PO_2_, partial pressure of oxygen; FIO_2_, fraction of inspired oxygen; CT, computed tomography. ^*^0.01 < *P* < 0.05; ^**^0.001 < *P* ≤ 0.01; ^***^P ≤ 0.001.

## Discussion

Despite the fact that the epidemic has been going on for half a year, our knowledge of this disease remains incomplete. The recurrence of the epidemic proves that we still need to pay more attention to the prevention and control of this disease.

This study reported 90 patients with confirmed COVID-19, of which 41 were clustered cases and 49 were sporadic cases. We found that patients in different groups showed no significant differences in demographics, clinical symptoms, and most laboratory findings, which was similar to the findings of Chen et al. (11). The most common symptoms were fever, cough, and expectoration, which is consistent with other studies (8, 12).

According to our findings, clustered cases had lower APACHE II scores on admission, fewer critical patients, and fewer patients receiving ICU care, mechanical ventilation, ECMO, ALSS, and CRRT compared with sporadic cases. The APACHE II score was widely used in the ICU to assess disease severity and mortality and was significantly higher in the severe group compared with the non-severe group (13). The fever duration and viral shedding in fecal specimens were shorter in clustered cases compared with sporadic cases. Viral shedding could be sustained until the patients' death (3). Delayed hospital admission could prolong the viral RNA shedding in the upper respiratory tract, and prolonged viral shedding could lead to worse clinical outcomes (14). Compared to sporadic cases, the time from illness onset to the end of intravenous corticosteroids use was shorter, and the possibility to develop ARDS and bacterial infections was reduced in clustered cases. For patients with progressive deterioration of oxygenation indicators, rapid progress in imaging and excessive activation of the body's inflammatory response, glucocorticoids would be used. Once the patients' laboratory findings got better, glucocorticoids would be gradually reduced and then stopped. The dosage of methylprednisolone changes from 20 to 80 mg/day according to each patient's condition. In a study of COVID-19, nine of 41 patients received corticosteroids, which could reduce the host lung inflammatory responses to avoid acute lung injury and ARDS (15). WHO highly recommended corticosteroids in patients with severe COVID-19 (16). However, it may also lead to delayed viral clearance and increased risk of secondary infection (17). ARDS is associated with pulmonary vascular permeability, increased lung weight, and loss of aerated lung tissue, and its 28-day mortality rate is up to 50% (18).

Above findings might suggest that patients with familial clustering had a milder disease and a better clinical outcome than sporadic cases, which is inconsistent with the findings of previous reports (8). This conclusion might be attributed to the fact that clustered cases had a more specific exposure history, shorter time from onset to diagnosis, and shorter time from onset to initiation of antiviral medication compared with sporadic cases. According to the Interim WHO Solidarity Trial Results, all four treatments evaluated (remdesivir, hydroxychloroquine, lopinavir, and interferon) had little or no effect on overall mortality, initiation of ventilation and duration of hospital stay in hospitalized patients (19). The 9th protocol published by National Health Commission of the People's Republic of China recommended that Paxlovid (300 mg PF-07321332 and 100 mg Ritonavir) or neutralizing monoclonal antibody therapy (1,000 mg BRII-196 plus 1,000 mg BRII-198) should be used to adults and adolescents with high-risk factors for progression from moderate to severe symptoms. Although, the result of a multinational clinical trial demonstrated that BRII-196 plus BRII-198 was safe, but not more effective than the placebo (20). Paxlovid did show efficacy in reducing the mortality and hospitalization rates in patients with COVID-19 without increasing the occurrence of adverse events (21). Like the previous report (22), our findings prove that the sooner we detect, diagnose, isolate and treat patients infected with SARS-CoV-2, the better clinical outcome patients have.

Epidemiological studies indicated that current social restriction measures for COVID-19 were effective in controlling the spread of the virus within large clusters, but might not be effective in small clusters and sporadic cases (23, 24). Enhanced management of contacts of COVID-19 cases and asymptomatic infected individuals is effective in reducing the potential of extensive dissemination of the virus within clusters (25). The results of these studies also confirm the need to enhance the early management of potential patients.

Although most of the clustered patients in this study had mild disease, some of them were still submitted to the ICU. To find out the difference in risk factors of ICU admission between clustered or non-clustered cases, we thoroughly compared the patients submitted to ICU and not submitted to ICU in two groups. We found that many of the same indicators differed between groups. We hypothesized that these indicators might be risk factors for patients admitted to the ICU, independent of whether they were clustered or not. IL-2 differed significantly between clustered cases admitted and not admitted to ICU. We indicated that IL-2 might be a specific indicator to identify the tendency of clustered cases to become critically ill. However, due to the small number of cases in this study, it might be incidental to draw conclusions. It needs large sample size to verify the hypothesis. There were some indicators that differed significantly only in sporadic cases, which might be related to the larger number of cases admitted to the ICU in it. If the number of cases was expanded, these indicators might also differ within clustered cases.

The study still has some limitations. First, the number of patients included in this study is relatively small, and the disease severity is relatively mild, which might have a selection bias and not fully reflect the characteristics of familial clustering. Second, patients may have memory bias when reviewing their exposure history, resulting in a reduced inclusion of clustered cases than those that actually occurred. Third, since the patients included in this study were all hospitalized in Zhejiang Province, regional bias may exist. Fourth, given that fewer patients received ICU care among the clustered cases, it may not allow a comprehensive exploration of risk factors for ICU admission among patients of a familial cluster. We would like to conduct new research about the clinical characteristics of a large cohort of clustered cases from each center of China.

In conclusion, compared with sporadic cases, clustered cases have milder disease severity, which may be closely related to the management of early detection, early diagnosis, early treatment and early isolation of COVID-19. Although the epidemic is relatively stable at present, another outbreak may occur. Therefore, it is recommended to strengthen the early detection, early diagnosis, early treatment and early isolation of the disease to prevent the epidemic from worsening again.

## Data availability statement

The original contributions presented in the study are included in the article/Supplementary material, further inquiries can be directed to the corresponding author.

## Ethics statement

The studies involving human participants were reviewed and approved by Ethics Committee of The First Affiliated Hospital, Zhejiang University School of Medicine. The patients/participants provided their written informed consent to participate in this study.

## Author contributions

Material preparation, data collection, and analysis were performed by WW, TD, DF, and JW. The first draft of the manuscript was written by XZ, WW, and DS. The revised manuscript was written by XZ and JN. All authors commented on previous versions of the manuscript, contributed to the study conception and design, read, and approved the final manuscript.
