# Supplementary material for: Clinical characteristics and clinical outcome of community clusters with SARS-CoV-2 infection
Source: Front Public Health. 2023 Jan 9;10:1010099. doi: 10.3389/fpubh.2022.1010099 (PMC9868852; doi:10.3389/fpubh.2022.1010099)
Supplement: Supplementary file 1 [file Table_1.docx]

**Clinical Characteristics and Clinical Outcome of Community Clusters with SARS-CoV-2 Infection**

Supplementary Table 1 Demographics and clinical characteristics of 90 patients with laboratory confirmed coronavirus disease 2019 infection.

|  | All patients (n=90) | Clustered cases (n=41) | Sporadic cases (n=49) | P-value |
| --- | --- | --- | --- | --- |
| Characteristics |  |  |  |  |
| Body mass index | 24.0±3.3 | 23.5±3.4 | 24.5±3.1 | 0.164 |
| Current smoking | 11(12.2%) | 5(12.2%) | 6(12.2%) | 0.994 |
| Current drinking | 7(7.8%) | 3(7.3%) | 4(8.2%) | 0.881 |
| Coexisting disorders |  |  |  |  |
| Diabetes | 12(13.3%) | 4(9.8%) | 8(16.3%) | 0.361 |
| Fatty liver | 12(13.3%) | 6(14.6%) | 6(12.2%) | 0.890 |
| Other diseases | 27(30%) | 12(29.3%) | 15(30.6%) | 0.890 |
| Signs and symptoms |  |  |  |  |
| Fever (≥37.4℃) | 75(83.3%) | 33(80.5%) | 42(85.7%) | 0.508 |
| Nausea | 5(5.6%) | 4(9.8%) | 1(2%) | 0.112 |
| Emesis | 2(2.2%) | 2(4.9%) | 0(0%) | 0.118 |
| Cough | 74(82.2%) | 32(78%) | 42(85.7%) | 0.343 |
| Expectoration | 43(47.8%) | 15(36.6%) | 28(57.1%) | 0.052 |
| Chest distress | 40(44.4%) | 16(39%) | 24(49%) | 0.344 |
| Abdominal pain | 2(2.2%) | 2(4.9%) | 0(0%) | 0.118 |
| Diarrhea | 11(12.2%) | 5(12.2%) | 6(12.2%) | 0.994 |
| Pharyngalgia | 5(5.6%) | 2(4.9%) | 3(6.1%) | 0.797 |
| Fatigue | 20(22.2%) | 8(19.5%) | 12(24.5%) | 0.572 |

Supplementary Table 2 Laboratory findings of 90 patients with laboratory confirmed coronavirus disease 2019 infection.

|  | All patients(n=90) | Clustered cases (n=41) | Sporadic cases (n=49) | P-value |
| --- | --- | --- | --- | --- |
| Laboratory findings |  |  |  |  |
| Red blood cell count (x10^12^/L) | 4.5(4.0-4.8) | 4.6(4.1-4.8) | 4.5(4.0-4.9) | 0.436 |
| White blood cell count (x10^9^/L) | 5.7(4.0-9.2) | 7.2(4.2-9.7) | 5.3(3.9-9.1) | 0.522 |
| Neutrophil count (x10^9^/L) | 4.2(2.6-7.8) | 5.3(2.6-8.1) | 3.9(2.7-7.5) | 0.676 |
| Lymphocyte count (x10^9^/L) | 0.8(0.5-1.2) | 0.8(0.5-1.4) | 0.8(0.6-1.2) | 0.897 |
| Haemoglobin (g/L) | 134.5(121.8-148.0) | 135.0(125.0-147.5) | 133.0(120.5-149.0) | 0.601 |
| Platelet count (x10^9^/L) | 183.5(144.8-237.8) | 191.0(148.5-243.0) | 181.0(142.5-238.5) | 0.579 |
| PCO_2_ | 35.5(33.7-39.1) | 37.3(33.6-39.7) | 34.9(33.6-38.2) | 0.065 |
| PO_2_/FIO_2_ | 274.6(166.9-164.7) | 289.1(232.5-431.3) | 260.5(164.7-378.5) | 0.149 |
| Lactic acid (mmol/L) | 1.7(1.3-2.2) | 1.6(1.3-2.1) | 1.8(1.3-2.4) | 0.923 |
| International normalized ratio | 0.98(0.94-1.03) | 0.97(0.94-1.01) | 0.99(0.95-1.05) | 0.059 |
| Active partial thromboplastin time (s) | 31.9(29.1-35.5) | 30.8(28.9-34.6) | 32.9(29.4-36.3) | 0.082 |
| D-dimer (mg/L) | 349.0(206.5-650.5) | 400.0(197.0-832.0) | 340.0(230.8-607.0) | 0.617 |
| Fibrinogen level (mg/dL) | 4.3(3.7-5.2) | 4.2(3.6-5.0) | 4.4(3.7-5.4) | 0.190 |
| Albumin (g/L) | 38.5(34.5-43.5) | 40.8(35.8-43.2) | 37.0(33.7-43.6) | 0.162 |
| Globulin (g/L) | 28.5(25.8-33.5) | 28.5(25.7-34.0) | 28.5(26.2-32.9) | 0.843 |
| Alanine aminotransferase (U/L) | 21.0(15.0-31.0) | 20.0(14.0-34.5) | 22.0(16.0-28.5) | 0.630 |
| Aspartate aminotransferase (U/L) | 22.0(16.8-30.3) | 22.0(18.5-29.5) | 22.0(16.0-35.5) | 0.706 |
| Alkaline phosphatase (U/L) | 61.5(54.0-78.3) | 62.0(54.0-77.5) | 61.0(51.5-80.0) | 0.935 |
| Gamma-glutamyl transferase (U/L) | 27.0(17.0-52.0) | 23.0(16.0-44.0) | 30.0(23.0-55.0) | 0.063 |
| Total bilirubin (μmol/L) | 11.1(7.9-16.7) | 10.1(6.8-15.7) | 11.6(8.4-18.1) | 0.257 |
| Direct bilirubin (mmol/L) | 4.7(3.3-7.6) | 4.3(3.1-6.8) | 5.3(3.5-8.5) | 0.113 |
| Creatinine (μmol/L) | 75.0(62.8-90.3) | 70.0(59.0-87.5) | 78.0(66.0-95.0) | 0.071 |
| Uric acid (μmol/L) | 243.0(188.3-296.8) | 248.0(215.5-341.0) | 221.0(172.0-286.0) | 0.137 |
| Triglyceride (mmol/L) | 1.2(0.9-1.7) | 1.2(0.9-1.9) | 1.2(0.9-1.7) | 0.348 |
| Total cholesterol (mmol/L) | 3.7(3.3-4.2) | 3.8(3.3-4.2) | 3.6(3.3-4.1) | 0.476 |
| High density lipoprotein (mmol/L) | 1.0(0.8-1.2) | 1.0(0.8-1.3) | 1.0(0.8-1.1) | 0.198 |
| Low density lipoprotein (mmol/L) | 2.0(1.7-2.4) | 2.0(1.7-2.4) | 2.0(1.7-2.5) | 0.973 |
| Glucose (mmol/L) | 7.0(5.3-8.7) | 6.5(5.0-8.3) | 7.5(5.4-9.2) | 0.238 |
| Potassium (mmol/L) | 3.8(3.5-4.2) | 3.8(3.5-4.1) | 3.9(3.5-4.3) | 0.624 |
| Sodium (mmol/L) | 139.0(137.0-141.0) | 139.0(137.5-141.0) | 139.0(136.0-141.5) | 0.563 |
| Procalcitonin (ng/mL) | 0.05(0.03-0.09) | 0.05(0.03-0.08) | 0.06(0.03-0.10) | 0.201 |
| C-reactive protein (mg/L) | 17.9(7.9-45.5) | 15.1(7.2-33.2) | 24.2(8.4-51.4) | 0.159 |
| Hydroxybutyrate dehydrogenase (U/L) | 207.5(165.8-272.8) | 196.0(156.5-252.5) | 231.0(175.5-294.0) | 0.056 |
| Creatine kinase (U/L) | 66.0(48.0-110.3) | 75.0(46.0-105.5) | 64.0(49.5-116.5) | 0.821 |
| Creatine kinase isoenzymes-MB (U/L) | 20.0(15.8-24.0) | 20.0(16.0-24.0) | 20.0(15.0-23.5) | 0.709 |
| Troponin I (ng/mL) | 0.003(0.002-0.008) | 0.004(0.002-0.008) | 0.003(0.001-0.008) | 0.809 |
| Interleukin-2 (pg/mL) | 1.0(0.8-1.5) | 1.0(0.7-1.3) | 1.0(0.8-2.1) | 0.144 |
| Interleukin-6 (pg/mL) | 18.6(8.0-50.5) | 14.3(5.7-33.3) | 24.1(8.7-71.0) | 0.052 |
| Interleukin-10 (pg/mL) | 4.2(2.7-7.3) | 3.8(2.1-7.7) | 5.0(3.0-7.2) | 0.140 |
| Interferon gamma (pg/mL) | 9.0(5.2-28.6) | 7.9(5.2-22.8) | 10.0(5.2-48.3) | 0.310 |
| Complement 4 (mg/dL) | 37.0(28.3-43.8) | 35.0(28.0-44.0) | 39.0(29.0-43.5) | 0.557 |
| Complement 3 (mg/dL) | 127.5(112.3-147.8) | 126.0(115.0-149.0) | 128.0(108.0-146.0) | 0.929 |
| Immunoglobulin A (mg/dL) | 207.5(160.5-280.3) | 206.0(170.0-281.0) | 209.0(152.0-283.0) | 0.651 |
| Immunoglobulin M (mg/dL) | 79.0(54.3-123.8) | 79.0(49.0-124.0) | 84.0(57.0-121.5) | 0.641 |
| Immunoglobulin G (mg/dL) | 1293.0(1021.0-1789.3) | 1290.0(1025.0-1984.0) | 1299.0(1005.5-1628.5) | 0.720 |

PCO_2_, partial pressure of carbon dioxide; PO_2_, partial pressure of oxygen; FIO_2_, fraction of inspired oxygen;
